# Unraveling the conumdrum: innovative technique to remove the stent from the displaced pancreatic duct in stenosis

**DOI:** 10.1055/a-2255-9168

**Published:** 2024-02-22

**Authors:** Junqian Chen, Shuting Wen, Xiaofeng Lin, Zhuodan Zhong, Yan Guo, Dongjing Zhou, Tianwen Liu

**Affiliations:** 1 74715 Department of Gastroenterology, The Second Affiliated Hospital of Guangzhou University of Chinese Medicine, Guangzhou, China; 2 74715 Department of Radiology, The Second Affiliated Hospital of Guangzhou University of Chinese Medicine, Guangzhou, China

Stent migration from the pancreatic duct (PD) is a relatively rare but serious complication 1 . If not treated in a timely way, it can lead to serious illnesses, such as recurrent pancreatitis, jaundice, pancreatic cysts, infection, etc. 2 . It is challenging to remove the displaced PD stent, especially in the presence of a PD stenosis, stent distortion, and adhesion to surrounding tissues 3 .

A 64-year-old woman underwent routine endoscopic retrograde cholangiopancreatography (ERCP) due to choledocholithiasis and biliary pancreatitis 3 years ago. The stone was successfully removed and a 5-Fr 5-cm PD stent was placed. Unfortunately, after 2 months, the stent had completely migrated into the PD. Removal using stone extraction balloons, baskets, snares, and foreign body forceps was unsuccessful. After suffering from recurrent pancreatitis for the past 3 years, another attempt was made to remove the PD stent.

During the operation, the guidewire was inserted into the proximal main PD but failed to advance to the cervical and caudal PD. Under the guidance of the pancreaticobiliary digital controller (eyeMAX; Micro-Tech Co Ltd, Nanjing, China), the stenosis and scar formation in the distal segment of the PD could be observed and the guidewire was placed through the stenotic segment in distal main PD. Dilation catheters and a balloon were used unsuccessfully in an attempt to dilate the narrowed PD. Finally, it was widened using the Cystotome cystoenterostomy needle knife (Cook Medical, Bloomington, IN, USA).

Using eyeMAX, the distal end of the PD stent was visualized. Removal of the PD stent with biopsy forceps and a basket was again unsuccessful. We then investigated a new method, and the PD stent was successfully removed using the guidewire orientation of dental floss and the foreign body forceps ( Fig. 1 , Video 1 ). The method that we report can safely remove the displaced PD stent in a stenosis without any complications.

**Fig. 1:**
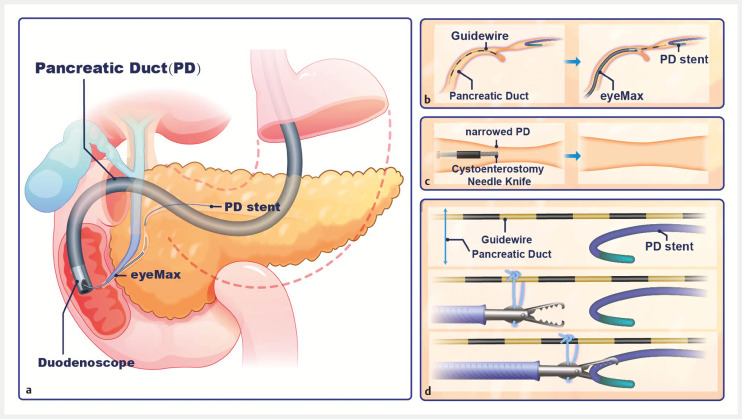
**a** Schematic diagram of the surgical procedure to remove an ectopic pancreatic duct (PD) stent in a narrowed PD. **b** Under the guidance of eyeMAX, the guidewire was threaded through the stenotic segment to the distal main PD. **c** Using the Cystotome cystoenterostomy needle knife, the narrowed PD was enlarged. **d** With the help of the dental floss traction guide and foreign body forceps, the PD stent can be successfully removed without significant trauma to the pancreas. Source: Shuting Wen and Mingyue Jin.

New method for recovering a displaced pancreatic duct stent in stenosis. Source for graphical illustration: Shuting Wen.Video 1

Endoscopy_UCTN_Code_CPL_1AK_2AD

## Notes

Endoscopy E-Videos https://eref.thieme.de/e-videos
*E-Videos* is an open access online section of the journal *Endoscopy* , reporting on interesting cases and new techniques in gastroenterological endoscopy. All papers include a high-quality video and are published with a Creative Commons CC-BY license. Endoscopy E-Videos qualify for HINARI discounts and waivers and eligibility is automatically checked during the submission process. We grant 100% waivers to articles whose corresponding authors are based in Group A countries and 50% waivers to those who are based in Group B countries as classified by Research4Life (see: https://www.research4life.org/access/eligibility/ ). This section has its own submission website at https://mc.manuscriptcentral.com/e-videos .
